# A voxel-level resting-state fMRI study on patients with alcohol use disorders based on a power spectrum slope analysis method

**DOI:** 10.3389/fnins.2024.1323741

**Published:** 2024-02-15

**Authors:** Xia Ruan, Zhiyan Song, Tingting Yu, Jun Chen

**Affiliations:** ^1^Department of Radiology, Renmin Hospital of Wuhan University, Wuhan, Hubei, China; ^2^Department of Radiology, Wuhan No. 1 Hospital, Wuhan, Hubei, China

**Keywords:** power spectrum slope, frequency band, functional magnetic resonance imaging, alcohol use disorder, spontaneous brain activity

## Abstract

**Background:**

Earlier neuroimaging investigations showed that abnormal brain activity in patients with alcohol use disorder (AUD) was frequency dependent. However, there is lacking of a comprehensive method to capture the amplitude of multi-frequency bands directly. Here, we used a new method, the power spectrum slope (PSS) to explore abnormal spontaneous activity of brain in patients with AUD.

**Methods:**

Thirty-three AUD patients and 29 healthy controls (HCs) enrolled in this study. The coefficient b and the power-law slope b’ were calculated and compared between two groups. We also used the receiver operating characteristic (ROC) curve to examine the ability of the PSS analysis to distinguish between AUD and HCs. We next examined the correlation between PSS difference in the brain areas and the severity of alcohol dependence.

**Results:**

Thirty AUD patients and 26 HCs were retained after head motion correction. The two metrics of PSS values increased in the left precentral gyrus in AUD patients. The area under the curve values of PSS differences in the specific brain area were respectively 0.836 and 0.844, with sensitivities of 86.7% and 83.3% and specificities of 73.1% and 76.9%. The Michigan Alcoholism Screening Test (MAST) and Alcohol drinking scale (ADS) scores were not significantly correlated with the PSS values in the specific brain area.

**Conclusion:**

As a novel method, the PSS can well detect abnormal local brain activity in the AUD patients and may offer new insights for future fMRI studies.

## 1 Introduction

It is a serious public health problem that alcohol is the most prevalent addictive substance and has high incidence and death rates. Alcohol use disorder (AUD) is characterized by persistent, excessive, uncontrollable consumption of alcohol and compulsive drinking behavior (Witkiewitz et al., 2019). Long-term heavy drinking can cause damage to the cardiovascular system, digestive system and immune system, especially the central nervous system, including Wernicke’s encephalopathy (WE), which can progress to Korsakoff’s syndrome (KS) if left untreated; hepatic encephalopathy (HE); central pontine myelinolysis (CPM); and Marchiafava-Bignami disease (MBD). In addition to its direct effects, heavy alcohol consumption increases the risk of seizures, stroke, and traumatic brain injury (Carvalho et al., 2019; Fritz et al., 2022).

Resting-state functional magnetic resonance imaging (rs-fMRI) is a non-invasive method, revealing the phenomenon of spontaneous neuronal activity at rest. One of its potential clinical applications is the exact localization of aberrant brain activity, which would aid in both the qualitative diagnosis and the direction of precise stimulation therapy, such as transcranial magnetic stimulation and deep brain stimulation (Zhao et al., 2022; Chang et al., 2023; Muller et al., 2023). Using rs-fMRI, (Abdallah et al., 2021) reported AUD patients existed abnormal connectivity between the cerebellum and both the frontoparietal executive control and ventral attention networks. Honnorat et al. (2022) suggested that functional connectivity between the anterior cingulate cortex and orbitofrontal cortex was lower in AUD patients, and functional connectivity between the anterior cingulate cortex and hippocampus was lower in AUD + HIV patients. The affected connections are associated with deficits in executive function, including increased impulsivity.

Among the myriad of rs-fMRI analysis methods, just few techniques have been developed for pinpointing aberrant brain activity and voxel-based meta-analyses. For instance, it has been proposed that amplitude of low-frequency fluctuation (ALFF) and its derivative fractional ALFF (fALFF) methods can be applied to analyze the local signal of rs-fMRI, which are widely used to evaluate spontaneous neuronal activity of certain areas under physiological or pathological conditions of the brain (Zang et al., 2007; Zou et al., 2008). A study reported (Liu et al., 2018) that brain differences caused by alcohol-related were found in the left precuneus with lower ALFF values, and these specific brain regions showed excellent discrimination between AUD and heathy controls (HCs) based on ALFF differences. Reduced fALFF in some regions may be useful in interpreting certain illnesses, such as enhanced sensitivity to identifying information connected to alcohol, which is typical of people with severe alcohol dependency (Deng et al., 2022). Alcohol-dependent patients showed abnormally increased spontaneous neural activity in the right cerebellum, which was more significant in alcohol-dependent patients with depression (Sun et al., 2023). These findings may support a targeted intervention in this brain location for alcohol and depressive disorder comorbidity.

A majority of studies examined conventional frequency band (Liu et al., 2018; Dai et al., 2023), but signal oscillations of the brain are integrated with multiple frequency bands (Buzsaki et al., 2013), and the result from one frequency band lacked frequency specificity. Different oscillation frequencies can be used to reflect various aspects of brain function (Zuo et al., 2010), suggesting that the study of fluctuations in brain signals in different frequency bands is also crucial for revealing the neural basis of the brain. Therefore, the consequence of frequency bands difference on functional brain connectivity was investigated in an increasing number of studies (Gimenez et al., 2017; Qi et al., 2018; Tang et al., 2023). The segmentation approach put forward by Zuo has been used in the majority of rs-fMRI research with multiple frequency bands (Zuo et al., 2010). Nevertheless, interpreting the results of group-level statistical analysis and the impact of varied frequency intervals may be challenging. There is a need for a comprehensive technique that can accurately capture the amplitude of multi-frequency bands. The distribution of the brain’s oscillations along the various frequency bands are various (Baria et al., 2011; He, 2011). Consequently, the slope of the brain signal’s power decline may be a good indicator of the distribution of brain oscillations (Zang et al., 2022).

Therefore, we used a novel method, the power spectrum slope (PSS), to explore the abnormal spontaneous activity of brain in AUD patients, then correlated these changes with clinical and neuropsychological data. The ability of PSS analysis to differentiate AUD from HCs was tested by receiver operating characteristic (ROC).

## 2 Methods

### 2.1 Subjects

The study was approved by Medical Ethics Committee of Renmin Hospital of Wuhan University, and followed the Helsinki Declaration. The consent of all subjects was obtained in writing.

Thirty-three right-handed male AUD patients and 29 age-, sex-, handedness-, and education-matched HCs were recruited from the primary-care outpatient department of Renmin Hospital of Wuhan University.

AUD patients were met the criteria of the Diagnostic and Statistical Manual of Mental Disorders: Fifth Edition (DSM-5) (the ICD-10-CM code: F10.10/F10.20). Inclusion criteria of AUD patients: a history of alcohol dependence for no less than 10 years; Alcohol drinking scale (ADS) score ≥ 14 and Michigan Alcoholism Screening Test (MAST) score ≥6 (Pilatti et al., 2017); no prior treatment history for AUD. HCs were those who had never or very seldom consumed alcohol (<1 standard unit per time) (Conley, 2001).

Subjects were excluded if they had any of the following: (1) an individual who exhibits psychotic symptoms or whose first-degree relative has been diagnosed with psychosis; (2) history of addiction to substances other than alcohol; (3) with organic brain disease or severe physical disease; (4) a history of cranial trauma, cranial surgery, brain tumor and coma; (5) people with previous seizures or a family history of epilepsy; (6) patients who have been treated with antipsychotic medication or who are receiving medication; (7) claustrophobia or any MRI contraindications.

The time interval between the last alcohol consumption and MR examination was 3 weeks for all subjects in this study to exclude the effects of acute alcohol intake.

### 2.2 Cognitive and alcohol level evaluation

Before undergoing MRI scans, all subjects underwent the Mini-Mental State Examination (MMSE) and Montreal Cognitive Assessment (MoCA); we only collected ADS and MAST scores from the AUD group to evaluate the level of alcohol dependence.

### 2.3 MRI acquisition

MRI scans were acquired using a 3.0 T MRI scanner (Discovery 750 W Silent MR, GE Healthcare, Milwaukee, WI). To avoid subject head movement artifacts during the experiment, the matching rubber soft plugs were used to make the head fixed. We also use soft foam ear plugs to reduce equipment noise. All subjects were asked to be quiet and relaxed, remain awake, keep their eyes closed and think of nothing in particular. Participants with lesions of brain were excluded from T_2_WI and T_2_-FLAIR images. High-resolution structural data were obtained in sagittal position: repetition time (TR) = 8.5 ms; echo time (TE) = 3.3 ms; field of view (FOV) = 240 mm × 240 mm; flip angle (FA) = 12°; matrix size = 256 × 256; slice thickness = 1.0 mm; no slice gap; and voxel size = 1 mm × 1 mm × 1 mm. The scanning parameters of fMRI data are as follows: TR = 2000 ms; TE = 25 ms; FOV = 240 mm × 240 mm; FA = 90°; slice thickness = 3.5 mm; no slice gap; matrix size = 64 × 64; interleaved axial slices = 40; and volumes = 240. Visual inspection of all MR images was carried out to make sure that none of the images included in the study had visible artifacts.

### 2.4 MRI data preprocessing and analysis

#### 2.4.1 Data preprocessing

MRI data was preprocessed using RESTplus tools embedded in the MATLAB software. The main preprocessing steps were as follows: (1) remove the first 10 time points because of inhomogeneities in the magnetic field and subjects’ maladaptive interferences with the environment; (2) slice timing; (3) realign, subjects with head movement >2.0 mm or 2.0° were excluded; (4) normalizing the data to the Montreal Neurological Institute (MNI) space by the technique of DARTEL (Ashburner, 2007); (5) smooth with 6 mm × 6 mm × 6 mm Gaussian kernel; (6) nuisance covariates regression, including Friston-24 parameters, white matter and cerebrospinal fluid.

#### 2.4.2 Power spectrum slope calculation

Following the aforementioned preprocessing steps, the Fast Fourier Transform was used to produce the power spectrum of each voxel time series. The frequency range was set to 0.01–0.25 Hz. Prior to fitting, the power of each voxel in brain was divided by the average amplitude, and the scale of power was normalized. We calculated the linear coefficient b and the power-law slope b’ according to the formula (Zang et al., 2022). For linear coefficient b, we used


(1) y=bx+a

For power-law fit b’, we used


(2) $$y=a^{\prime }x^{b^{\prime }}$$

where *y* is the normalized amplitude of the signal power after FFT, *x* is the corresponding frequency bin (e.g., from 0.01 to 0.25 Hz), b is the linear coefficient and b’ is the power-law slope (Zang et al., 2022). The larger negative values of these two metrics indicated steeper decay of power from low to high frequencies. By using the Z-transform, we obtained PSS z-score maps for each subject.

### 2.5 Statistical analysis

#### 2.5.1 Clinical information

Baseline clinical information was compared between the two groups using SPSS 26.0. Demographic and clinical data for both groups were tested by the Shapiro–Wilk test and independent samples *t*-tests. The threshold was *p* < 0.05.

#### 2.5.2 PSS analysis

We compare spatial distribution maps of PSS values within groups. Then we conducted a two-sample *t*-test on the two metrics (coefficient b and power-law slope b’) to analyze PSS differences. The statistics were corrected with Alphasim method (voxel *p* < 0.001, cluster *p* < 0.05).

#### 2.5.3 ROC curve and correlation analysis

To determine if the PSS values might be used as a biological marker for differentiating AUD patients from HCs, the performance of the PSS on distinguish between AUD and HCs was examined by ROC curve.

To investigate the specific relationships between PSS values and MAST/ADS scores, the correlation between altered PSS values and the severity of alcohol dependence was evaluated. The significantly different areas from the PSS analysis described above were extracted and relationships between PSS values in these areas and the patients’ MAST/ADS scores were analyzed. Spearman’s correlation coefficient was calculated.

## 3 Results

### 3.1 Clinical information

Six participants (3 AUD patients and 3 HCs) were excluded following correction for head motion. Finally, 30 AUD patients and 26 HCs were included in this research. Clinical information of the two groups were shown in Table 1. In terms of gender, age, education, and handedness, no statistically significant difference was found. Nonetheless, the MMSE and MoCA scores of the AUD group were considerably lower (*p* < 0.05) than the HCs group.

**Table 1:** Demographic and clinical characteristics.

Characteristics	AUD (*n* = 30)	HCs (*n* = 26)	*p*-value
Age (years)	53.03 ± 5.49	49.96 ± 6.08	0.052
Gender (male/female)	30/0	26/0	-
Education level (years)	11.17 ± 2.78	10.35 ± 3.07	0.299
Handedness (R/L)	30/0	26/0	-
MoCA	25.17 ± 2.83	26.65 ± 1.98	0.002
MMSE	26.73 ± 1.86	28.19 ± 1.34	0.002
MAST	8.83 ± 3.36	-	-
ADS	17.03 ± 3.54	-	-

Values are expressed as means ± standard deviations or frequencies. *p* < 0.05 was considered statistically significant. AUD, alcohol use disorder; HCs, healthy controls; MoCA, Montreal Cognitive Assessment; MMSE, Mini-Mental State Examination; MAST, Michigan Alcoholism Screening Test; ADS, Alcohol Drinking Scale.

### 3.2 PSS analysis

**Figure 1:**
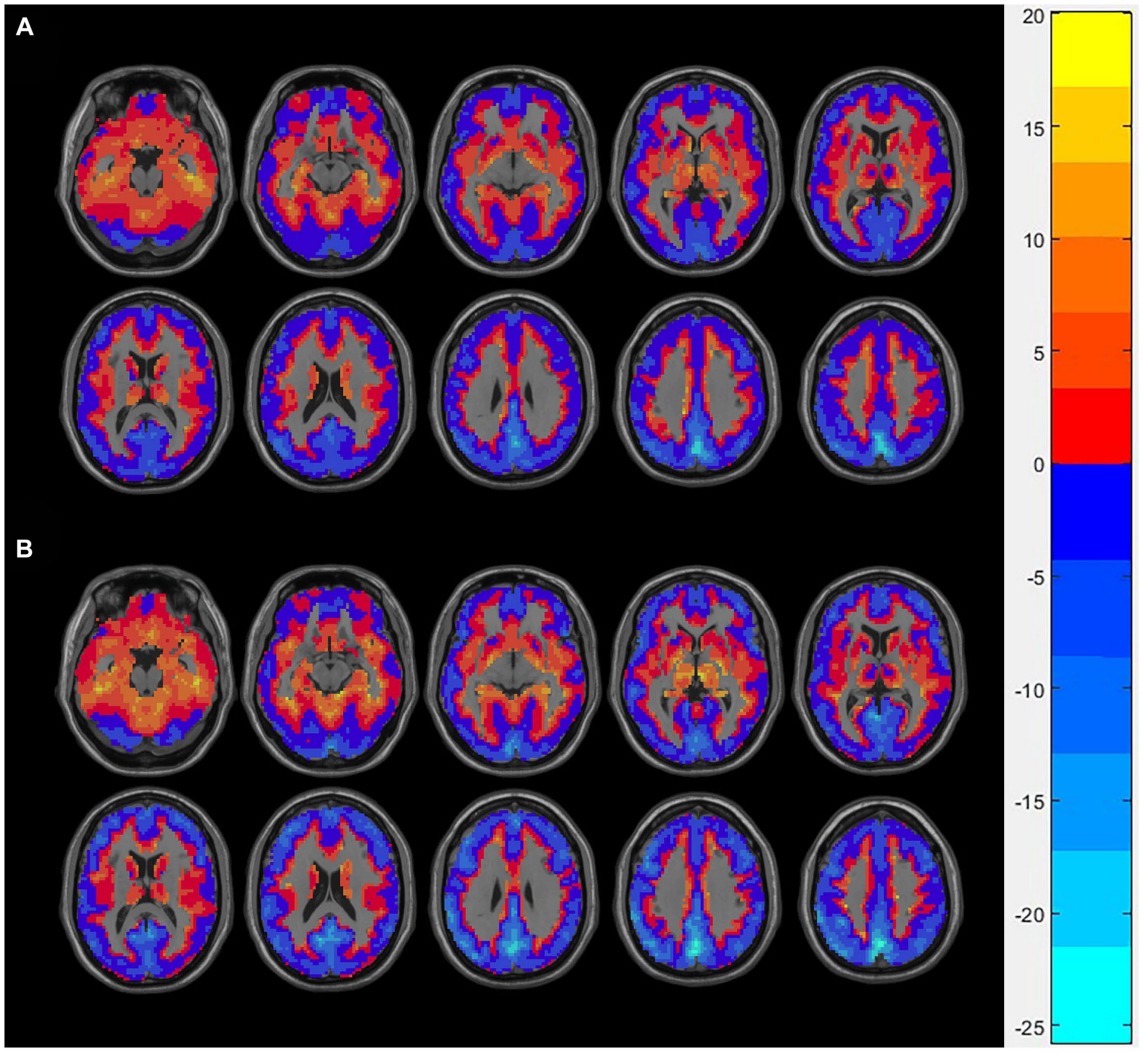
The spatial distribution of Z-transformed PSS (linear coefficient b) in the AUD group **(A)** and the HCs group **(B)**, where warm and cold color, respectively, represents the area with high and low PSS values. PSS, power spectrum slope; AUD, alcohol use disorder; HCs, healthy controls.

We showed the spatial distribution maps of PSS values (linear coefficient b) in AUD group and control group in Figure 1. The bulk of the cerebral cortex in both the AUD and HCs groups displayed lower PSS values, particularly in the visual region with a steeper slope than the whole-brain average PSS. White matter was where higher PSS values were discovered.

**Figure 2:**
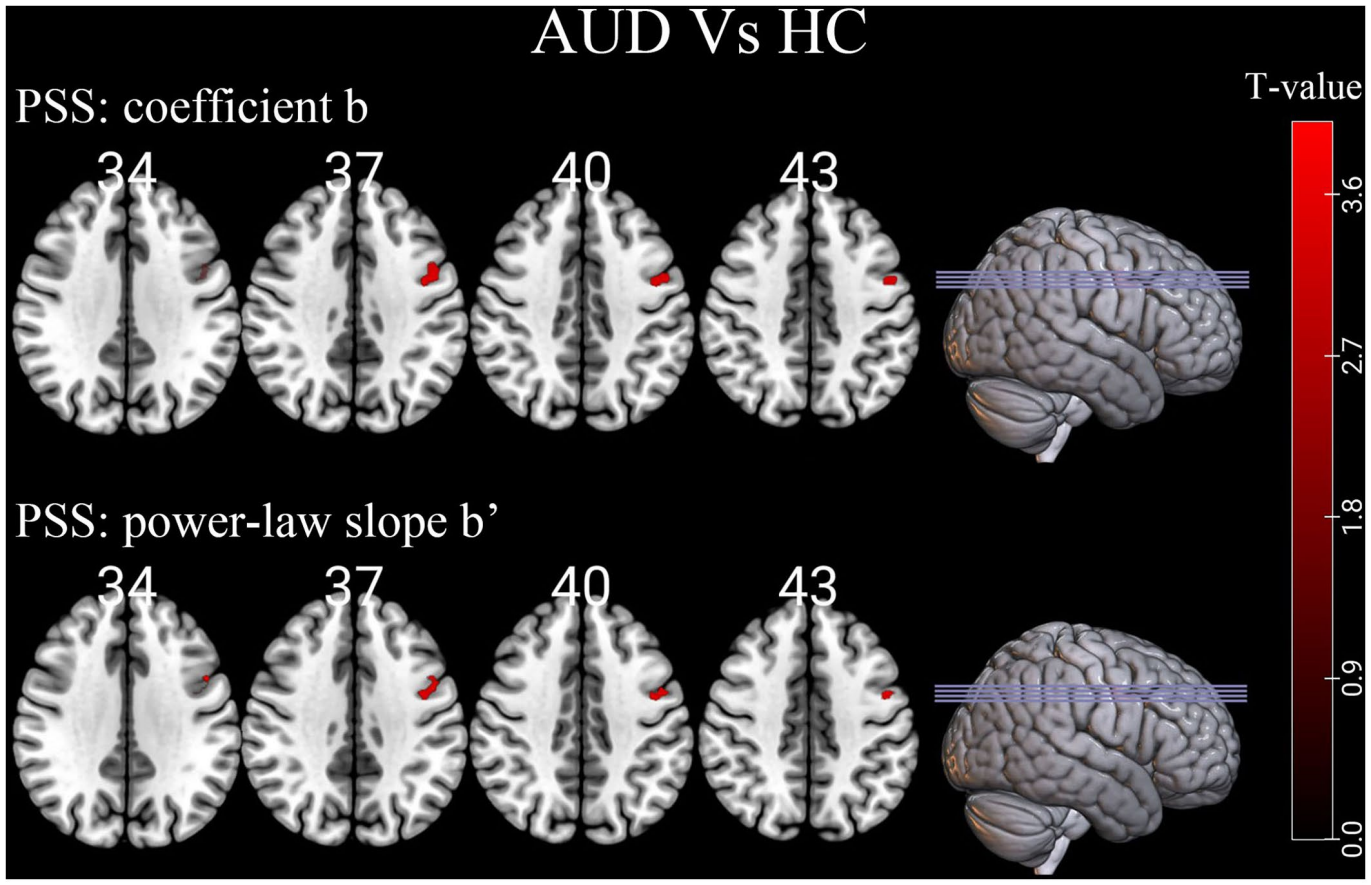
Differences of individual-level PSS (Z-transformed coefficient b and power-law slope b’) in brain regions between AUD and HCs. Red denotes higher PSS values in AUD patients than HCs. PSS, power spectrum slope; AUD, alcohol use disorder; HCs, healthy controls.

Compared to HCs group, the linear coefficient b and the power-law slope b’ in the left precentral gyrus (PreCG) increased in the AUD group (Table 2; Figure 2).

**Table 2:** The PSS difference between AUD and HCs.

Brain regions	BA	Cluster size	Peak MNI coordinates			*T* value
			X	Y	Z	
*PSS: coefficient b*						
L-precentral gyrus	6	27	−48	6	36	4.2180
*PSS: power-law slope b’*						
L-precentral gyrus	6	22	−45	−3	42	4.2243

PSS, power spectrum slope; MNI, Montreal Neurological Institute; BA, Brodmann area; AUD, alcohol use disorder; HCs, healthy controls; L, left.

### 3.3 ROC curve

**Figure 3:**
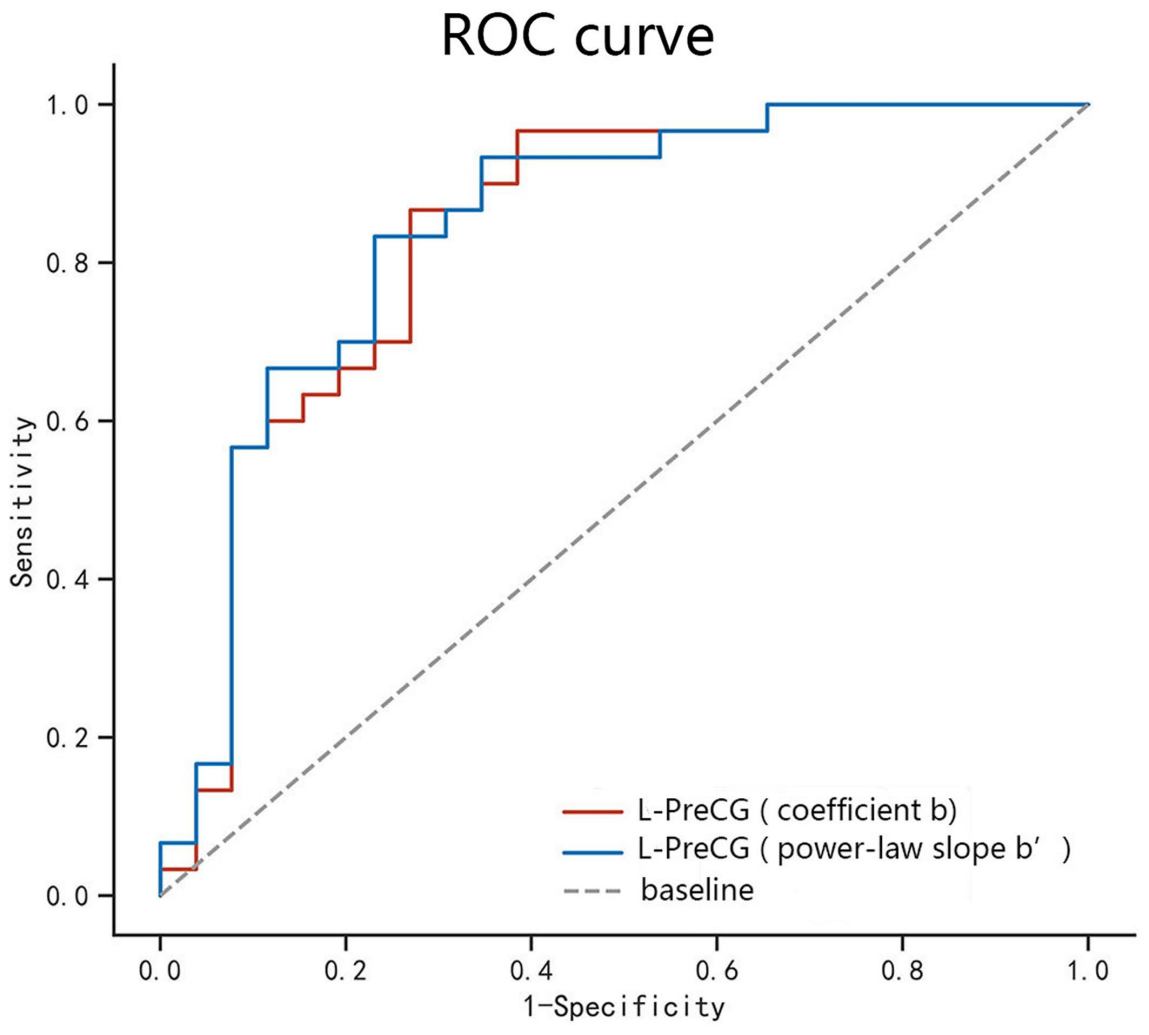
ROC curve analysis of the PSS difference in the regional brain areas. The diagnostic efficiency of the coefficients b and power-law slopes b’ in the left PreCG was, respectively, 0.836 and 0.844, with sensitivities of 86.7% and 83.3% and specificities of 73.1% and 76.9%. ROC, receiver operating characteristic; PreCG, precentral gyrus; L, left.

According to our research, the area under the curve values of PSS differences in the left PreCG was, respectively, 0.836 and 0.844, with sensitivities of 86.7% and 83.3% and specificities of 73.1% and 76.9% (Table 3; Figure 3).

**Table 3:** ROC curves for distinguishing PSS in PreCG of AUD patients from that of HCs.

Brain area	AUC	Sensitivity, %	Specificity, %	Cutoff point
L-PreCG (coefficient b)	0.836	86.7%	73.1%	−0.623
L-PreCG (power-law slope b’)	0.844	83.3%	76.9%	−0.547

ROC, receiver operating characteristic; AUC, area under the curve; PSS, power spectrum slope; AUD, alcohol use disorder; HCs, healthy controls; PreCG, precentral gyrus; L, left.

### 3.4 Correlation analysis

The MAST or ADS scores were not significantly correlated with the PSS values (coefficient b and power-law slope b’) in the left PreCG.

## 4 Discussion

In this study, we compared linear coefficient b and power-law slope b’ of AUD patients and HCs by using PSS method. PSS differences in the particular brain area could well distinguish AUD patients from controls with good AUC, sensitivities and specificities.

As an example, we displayed the spatial distribution of Z-transformed PSS images with linear coefficient. Z-PSS values well displayed gray-matter and white-matter boundaries, respectively, corresponding to negative and positive values. In addition, the gray matter voxels showed greater decline of signal strength than the white matter (larger absolute slope than the global mean), while the greatest power decay was found in the visual brain region.

The rs-fMRI signal’s amplitude declines with higher frequency bins in the frequency domain, however the decreasing rate differed between the AUD group and HCs group. As demonstrated in this study, no regions decayed more in the AUD group while signal power of the left PreCG decayed less in the AUD group than that in the HCs group. Both the linear coefficient b and the power-law slope b’ can detect the difference. The differences in PSS between the AUD and HCs groups may reflect different mechanisms of cortical physiological states. Larger power-law exponent indicates a higher time-lagged autocorrelation, suggesting that the past dynamics of the system has a greater influence on its future dynamics, i.e., the system has more long-range memory, while smaller power-law exponent indicates that the system has less temporal redundancy and is more efficient in online information processing (Eke et al., 2002; He, 2011). Therefore, the region of “shallower” power decay in the AUD group indicating more redundant neural processing of information.

In this study, alcohol-triggering brain differences mainly located in the left PreCG (Brodmann’s area [BA] 6), which was generally consistent with earlier work of our team and previous research (Tu et al., 2018; Ruan et al., 2023). The PreCG is in the frontal lobe of the cerebral cortex and is the primary motor center, involved in the integration of information related to the sensory, motor, attention, and reward circuits (Stinear et al., 2009; Gardini and Venneri, 2012; Squeglia and Gray, 2016). Our finding of altered PSS in the left PreCG region of AUD patients further enriched the evidence that chronic alcohol consumption may lead to functional changes in the PreCG that affect sensory, motor, and attentional functions as well as brain reward mechanisms. A Reho study showed that compared with healthy controls, the left PreCG (BA 6) of alcohol-dependent patients exhibited a significantly higher ReHo area, and higher Reho values in the supplementary motor area (BA 6) may be a brain compensatory mechanism or hyperactivation (Tu et al., 2018). Alcohol-dependent individuals often exhibit compulsive drug-seeking behaviors, which may be related to abnormal brain activity in the supplementary motor area (BA 6), showing an excessive motor response in human addiction processing. The resting-state degree centrality analysis method also found male AUD patients had significantly higher degree centrality values in the left PreCG (BA 6) than HCs, and they also found a strong unilateral lateralization in male AUD patients, showing that the number of different degree centrality values in the left side of the brain regions of AUD patients was higher than in the right side, and that this laterality was also reflected in the total number of voxel volumes with different degree centrality values in the brain regions (Luo et al., 2017). The PreCG are important and specific in impeding episodic memory (Fama et al., 2021), and larger power-law exponent suggests more long-range memory (Eke et al., 2002), which also well explains the PSS change in the left precentral gyrus of AUD patients in our study. Aberrant functional connectivity between the PreCG and left anterior insula was associated with processing of stressful experiences (Goodman et al., 2022). According to our research, the left PreCG was functionally implicated in saccade execution and may play a role in determining an individual’s antisaccade cost (Jin et al., 2022).

This study also investigated the feasibility of PSS method in differentiation of abnormal spontaneous activity of brain in AUD patients from healthy volunteers. ROC curve revealed good AUC values of the specific brain area, and further diagnostic analysis demonstrated that the specific brain area alone discriminated the AUD patients from the healthy subjects with high degree of sensitivities and specificities. The diagnostic efficiency of the coefficients b and power-law slopes b’ in the left PreCG was, respectively, 0.836 and 0.844, with sensitivities of 86.7 and 83.3% and specificities of 73.1 and 76.9%. The diagnosis performance of PSS was good. Correlation analysis showed that the MAST or ADS scores were not significantly correlated with the PSS values in the specific brain area. In conclusion, for the time being, the present study fills the research gap of the PSS method in the study of brain activity abnormalities in patients with AUD. Our results confirmed that this approach is feasible and offers new insights for AUD studies.

There were some limitations in our study. Firstly, the sample size is small. However, rs-fMRI studies with more than 16 subjects per group are acceptable (Friston, 2012). Secondly, the results may not apply to female AUD subjects due to the fact that only men were included. Thirdly, this study was only preliminary to determine if there were differences in cognitive functioning between AUD and HCs groups, so more complex neuropsychological assessments were not conducted. More detailed neuropsychological assessments will be further explored in future studies. Finally, this study is cross-sectional, and dynamic changes of PSS in AUD patients could not be observed at various stages, which may require further longitudinal studies to address. Our results confirmed that PSS method may be a useful biological indicator for the detection of regional brain activities in patients with AUD, and offers new insights for AUD studies.

## 5 Conclusion

Our study demonstrated that the linear coefficient b and power-law slope b’ of the PSS method could well detect abnormal local brain activity in the AUD patients and may become a complement method in detecting localized spontaneous activity.

## Data availability statement

The datasets presented in this article are not readily available because the raw dataset is not publicly available due to the inherently identifiable nature of the data. Requests to access the datasets should be directed to JC. Requests to access the datasets should be directed to whuchenjun@163.com.

## Ethics statement

The studies involving humans were approved by Ethics Committee of the Renmin Hospital of Wuhan University. The studies were conducted in accordance with the local legislation and institutional requirements. The participants provided their written informed consent to participate in this study.

## Author contributions

XR: Writing – original draft, Writing – review & editing, Conceptualization, Data curation, Formal analysis, Investigation, Methodology, Software, Validation, Visualization. ZS: Writing – original draft, Data curation, Investigation. TY: Writing – original draft, Project administration, Validation. JC: Funding acquisition, Project administration, Resources, Supervision, Writing – review & editing.
